# Influence of Sodium and Potassium Chloride on Rennet Coagulation and Curd Firmness in Bovine Milk

**DOI:** 10.3390/foods12122293

**Published:** 2023-06-07

**Authors:** Fabijan Oštarić, Samir Kalit, Ino Curik, Nataša Mikulec

**Affiliations:** 1 Department of Dairy Science, Faculty of Agriculture, University of Zagreb, 10000 Zagreb, Croatia; fostaric@agr.hr (F.O.); skalit@agr.hr (S.K.); 2 Department of Animal Science, Faculty of Agriculture, University of Zagreb, 10000 Zagreb, Croatia; icurik@agr.hr

**Keywords:** sodium, potassium, chloride, substitution, coagulation properties, bovine milk

## Abstract

One of the salting methods in cheese production implies salting the milk before coagulation used in making Domiati-type cheeses and a variety of autochthonous “Lički Škripavac” cheese. The most used sodium replacer is potassium. This study investigated the influence of different added salt concentrations (1%, 1.5%, and 2%) and NaCl to KCl ratios (100%, 50:50%, 25:75%) on the rennet coagulation and curd firmness in bovine milk. The milk coagulation parameters were determined with a computerized renneting meter, Lactodinamograph. The results showed significant interactions between the salt concentrations and NaCl to KCl ratios (*p* < 0.0001, α = 0.05) by prolonging the beginning of coagulation (10–20 min) and curd firming rate (1–5 min) by an increase in salt concentration for all treatments. The 50:50 treatment values (RCT, k_20_, a_30_, a_60_, a_max_) were closest to the control (without salt) and had the best results among all treatments in the lower (1%) and medium (1.5%) salt concentration (*p* > 0.0001, α = 0.05) while in the highest salt concentration (2%) the treatment effect was nonsignificant (*p* > 0.05). These results should help future studies make a lower sodium product appealing to consumers without losing quality.

## 1. Introduction

Since ancient times, sodium chloride (NaCl) has been used for food flavoring and preservation. It has always been closely related to different aspects of human history [1]. Sodium chloride accounts for 95% of sodium intake [2], with table salt consisting of 40% sodium and 60% chloride by weight [3]. Average intake exceeds current guidelines and recommendations from WHO [4] of 2.300 mg/day and EFSA [5] of 2.000 mg/day in the EU, respectively. For this reason, new dairy products with lower sodium content are investigated. According to van Buren et al. [6], food producers need to ensure that products with lower sodium content remain appealing to consumers, so sodium reduction in food products should not lead to a loss in quality. 

The leading method for reducing sodium in food products is substitution with potassium (i.e., potassium chloride, KCl), the most used salt replacer [6]. Current EU recommendations for potassium intake are 3500 mg/day for adult men and women [7]. Studies explain its wide use because it gives a salty taste [3,8], improves shelf life due to its antimicrobial property [3,9], and even lowers blood pressure in people with hypertension with a 24% lower chance of stroke [10]. Potassium chloride is widely used for sodium replacement in cheese production due to its salty taste and antimicrobial activity. Studies show sodium replacement with up to 50% potassium in cheese is possible without effect on sensory characteristics [3,11,12].

**Figure 1:**
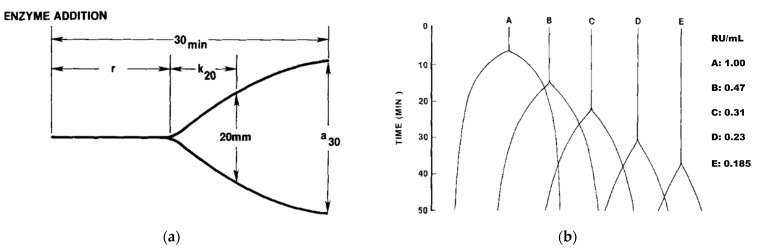
Schematic representation of (**a**) milk coagulation properties (r = RCT, k_20_, a_30_); (**b**) lactodynamographic curves at various rennet concentrations with quality classes (A–E); RU/mL—rennet concentrations; from McMahon & Brown, (1982) [16].

Cheese quality is affected by its chemical composition, microbiological quality, and production process [13]. According to Troch et al. [14], to optimize the transformation of milk into cheese, it is necessary to investigate and understand milk coagulation as an essential parameter with the factors affecting it. The most common approach to studying milk coagulation is to monitor changes in milk viscosity at fixed temperature after the addition of rennet [15,16] with Formagraph or Lactodinamograph. The main milk coagulation properties (MCP) studied are rennet coagulation time (RCT, min) which is the time needed from rennet addition to the start of the coagulation, time to reach curd firmness (CF, mm) of 20 mm (k_20_, min), CF 30 min after rennet addition (a_30_, mm) [15,17,18]. Newer studies include prolonged observation and parameters a_45_ and a_60_ (CF 45 and 60 min after rennet addition) with curd firmness modeling as a function of time to include non-coagulating milk samples [19]. Figure 1 shows diagrams of milk coagulation properties, coagulation, and firmness as a function of time made by McMahon & Brown (1982) [16].

One of the salting methods in cheese production implies salting the milk before coagulation used in Domiati-type cheeses [20,21] and a variety of autochthonous “Lički Škripavac” cheese [22]. Studies on lowering sodium in cheese investigated the substitution of mineral salt to retain saltines and the preservation effect in cheese [23] because simply lowering sodium negatively affected sensory properties and microbiological quality [24,25,26]. Studies on partially replacing sodium with potassium stated no significant chemical, microbiological or sensory differences [27,28,29,30].

Zhao & Corredig [31] reported that adding NaCl influenced both primary and secondary rennet coagulation stages, whereas gelation time increased. Adding NaCl to milk decreased rennet clotting activity and gel firmness [32,33]. Potassium negatively correlated with rennet clotting time (RCT, min) [34]. Stocco et al. [35] report that potassium naturally found in milk seemed not actively involved in coagulation and cheese-making. Finally, the decrease in sodium can be differently approached according to cheese type and technology [26], so its effect on the coagulation process must be understood better.

This research aims to understand how added sodium and potassium chloride to the milk influence rennet coagulation, curd firmness, and other milk coagulation properties (MCP) by studying four treatments with different NaCl to KCl ratios and three different treatment concentrations to find the optimal sodium-to-potassium ratio in cheese with total sodium lowered. The optimal sodium-to-potassium ratio should benefit the production process to keep the same or improve the final product quality. 

## 2. Materials and Methods

### 2.1. Milk Sampling and Experimental Design

A bulk milk sample from a Simmental breed cow in Central Croatia, near Zagreb, was taken for the experiment. The milk sample was taken in the morning of July 2021 directly from the bulk tank on the farm and transported to the laboratory in a portable cooler with ice packs. It was stored in the laboratory fridge (+4 °C) until treatments were added later the same day and analysis made.

The experiment was designed to have four treatments based on the NaCl to KCl ratio (T1) presented in Table 1 and three treatments (T2) based on different concentrations of salts (1 = 1.0%, 2 = 1.5%, 3 = 2.0%). The raw milk was divided into five identical samples representing treatments A, B, C, D, and E. Each sample from treatment T1 was then divided into three identical subsamples (1, 2, and 3) representing treatment concentrations (T2). 

Before dividing the milk, samples were taken for chemical, physical, and microbiological analysis. After dividing, the treatments were added to the milk based on the experimental design stated previously. The samples were then incubated in a water bath at 37 °C for 60 min before the analyses with constant mixing to dissolve the salts properly.

### 2.2. Chemical, Physical, and Microbiological Properties

Milk fat, protein, total solids, non-fat solids, and lactose were determined on the Milkoscan FT3 (Foss, Hilleroed, Denmark) according to ISO 9622:2013 [36]. Somatic cell count was determined on the Fossomatic Minor (Foss, Hilleroed, Denmark) with the enumeration of somatic cells, fluoro–opto–electronic method according to ISO 13366-3 [37]. The total bacterial count (TBC) was determined with the horizontal method for the enumeration of microorganisms—colony counts at 30 °C by the pour plate technique, ISO 4833-1:2013 and ISO 21187:2021 [38,39] in plate count agar with skim milk (Biolife Italiana S.r.l, Milano, Italy). Milk acidity was measured with pH meter Seven Multi (Mettler-Toledo GmbH, Greifensee, Switzerland) according to the manufacturer’s instructions.

### 2.3. Determination of Milk Coagulation Properties (MCP) 

Coagulation properties were determined with a mechanical Lactodinamograph or formagraph (MA.PE SYSTEM SRL, Firenze, Italy) as described by Bittante (2011) [15]. Four repetitions of each treatment combination were analyzed, and pendula were calibrated before each session. The traditional MCP parameters determined and later used in statistical analyses were RCT, k_20_, a_30_, a_60_, and a_max_, respectively. As previously described, rennet coagulation time (RCT, min) is the time needed from rennet addition to the start of the coagulation, k_20_ is the time required to reach curd firmness (CF, mm) of 20 mm (k_20_, min), a_30_ is the curd firmness 30 min after rennet addition (a_30_, mm), a_60_ is the curd firmness 60 min after rennet addition, and maximum curd firmness (a_max_, mm).

#### 2.3.1. Rennet Solution Preparation

The powdered rennet used for analysis was of natural, animal origin (Caglificio Clerici SPA, Cadorago, Italy) with a strength of 890 IMCU/g and 70:30 chymosin to pepsin ratio. The rennet was diluted to 0.29% (*w*/*v*) in distilled water to 0.0513 IMCU/mL before the session.

#### 2.3.2. Lactodinamograph Analysis

The samples were incubated in a water bath at 37 °C for 60 min. After incubation, pH was measured with pH meter Seven Multi (Mettler-Toledo GmbH, Greifensee, Switzerland), four 10 mL repetitions of every sample were put in the setting block, and 200 µL of rennet solution was added. The analysis was set for 60 min, and the results were later retrieved for statistical analysis.

### 2.4. Statistical Analysis 

Two models were used for the estimation of variance and the effect of the treatments on the coagulation properties. 

The primary model to investigate a possible interaction between treatments, an interaction model with milk coagulation properties as dependent variables, was used:

(1) $$Y_{ijk}=\mu +{T1}_{i}+{T2}_{j}+{\left(T1*T2\right)}_{ij}+e_{ijk}$$

where $Y_{ijk}$ = dependent variables tested with the model (RCT, k_20_, a_30_, a_60_, a_max_); *μ* = overall mean; ${T1}_{i}$ = effect of the level i of the factor *T*1; ${T2}_{j}$ = effect of the level *j* of the factor *T*2; ${\left(T1*T2\right)}_{ij}$ = effect of the interaction of the level *i* of the factor *T*1 and level *j* of factor *T*2; $e_{ijk}$ = residual. 

A standard linear model was later used to additionally investigate the effect of NaCl and KCl on observed coagulation parameters inside every level of treatment concentration (*T*2):

(2) $$Y_{ij}=\mu +{T1}_{i}+e_{ij}$$

where $Y_{ij}$ = dependent variables tested with the model (RCT, k_20_, a_30_, a_60_, a_max_); *μ* = overall mean; ${T1}_{i}$ = effect of the level *i* of the factor *T*1; $e_{ijk}$ = residual. 

The statistical analyses were all conducted in SAS 9.4 (Cary, NC, USA: SAS Institute Inc., [40]). Descriptive statistics were calculated using PROC MEANS. PROC GLM was used for the one and two-way ANOVA, Duncan’s new multiple range test (MRT), LSMeans, and plots.

## 3. Results

### 3.1. Physicochemical and Microbiological Milk Quality

The physicochemical and microbiological results of the milk sample are represented in Table 2. 

### 3.2. The Two-Way ANOVA and Interactions between Treatments (Interaction Model)

**Figure 2:**
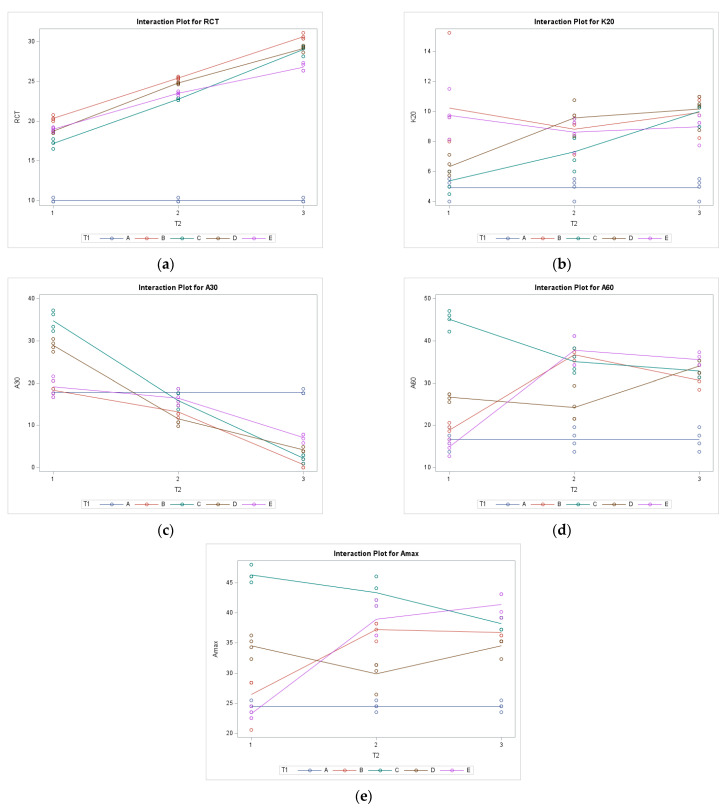
Interaction plots for (**a**) RCT—rennet coagulation time, (**b**) k_20_—time to reach curd firmness (CF) of 20 mm, (**c**) a_30_—curd firmness 30 min after rennet addition, (**d**) a_60_—curd firmness 60 min after rennet addition, and (**e**) a_max_—maximum curd firmness in the interaction model; *x*-axis = salt concentration T2, *y*-axis = observed parameter; A—control, B—100% NaCl, C—50:50, D—25:75, E—100% KCl.

The results from the two-way ANOVA show significant interaction (*p* < 0.0001, α = 0.05) between treatments T1 and T2. The model explains more than 95% of the variance for the parameters RCT (R^2^ = 0.99), a_30_ (R^2^ = 0.98), a_60_ (R^2^ = 0.96), a_max_ (R^2^ = 0.95), and 78% for the parameter k_20_ (R^2^ = 0.78). The model shows how salt concentration changes affect NaCl to KCl ratios and influence MCP-s. Figure 2 shows interaction plots from the model.

### 3.3. The One-Way ANOVA of NaCl to KCl Ratio Effects (T1) inside Every Level of Treatment Concentration (T2)

The results from the one-way ANOVA show different NaCl to KCl ratios significantly (*p* < 0.0001, α = 0.05) influencing rennet coagulation and curd firmness. After removing interaction between treatments, the model still explains a very high percentage of the variance. A growth in R^2^ for the parameter k_20_ is observed, suggesting greater salt concentration’s effect on the curd firming rate. Table 3 represents means and Duncan’s MRT with R^2^ (coefficient of determination) and CV (coefficient of variation) for every MCP parameter through both treatments (T1 and T2). 

## 4. Discussion

### 4.1. Physicochemical and Microbiological Milk Quality

Overall, milk quality was satisfactory for the experimental study. The pH value of 6.55 was within the limits of Croatian regulations (NN 136/2020) [41]. The milk fat was 0.59% (4.1%) and protein 0.06% (3.4%) lower than the average for the Simmental breed in Croatia [42] but within Croatian regulations (NN 136/2020) [41]. Somatic cell count and microbiological quality followed the EU regulation 853/2004 [43] and Croatian regulation (NN 136/2020) [41] but showed affiliation in the second quality class. 

The lactose percentage (LP) was significantly lower than the average of 4.28–4.70 [44,45]. According to the literature and available research, there are several reasons for lower LP. Lactose synthesis and concentration in cow milk are affected mainly by udder health and cow’s energy balance and metabolism [45]. Studies show that increases in somatic cell count (SCC) and total bacterial count (TBC) are directly associated with lower lactose content, with emphasis on mastitis-causing strains of bacteria having more impact on lactose fermentation [46,47]. Najera et al. [48] state that a decrease in milk fat and protein percentage follows this effect because of lipolysis and proteolysis caused by the bacteria. Costa et al. [45] reported that LP strongly depends on circulating blood glucose levels, so milk from subketotic and undernourished cows tends to show lower LP. Some studies report significantly greater LP in cows fed with high dietary concentrate [49] and a significant 15% decrease in LP in the milk of cows fed a low-caloric density diet [50]. Since the samples were taken in July, most likely a combination of heat stress where cows increased energy expenditure to cooling, a low-quality diet, and increased TBC resulted in lower lactose levels. 

Croatian regulations [41] state that the acidity should not be lower than pH 6.5 with an adverse reaction to the addition of 72% ethanol (no induced gelation). Although measured milk acidity was inside requirements, it might have influenced researched parameters RCT and k_20_ regarding faster coagulation. For example, the coagulation for the control group A started 10 min faster than the average 12–19 min, depending on the breed and milk acidity [51,52,53]. Research confirms the influence of milk acidity on the beginning of the coagulation, curd firming rate, and overall curd firmness. Formaggioni et al. [51] reported a strong negative correlation between milk acidity and RCT (r = −0.74) and k_20_ (r = −0.50). Najera et al. [48] report a significant effect of milk acidity on milk coagulation properties in the 6.8–6.0 pH range. Awad et al. [33] reported no significant effect of pH on RCT and k_20_ in the pH range of 6.6–6.4. They reported a significant effect with pH < 6.4 in producing Domiati-type cheese.

According to studies, milk acidity could have influenced the beginning of the coagulation (RCT) and curd firming (k_20_). Still, this effect was carried over to all the samples having the same influence on the research results.

### 4.2. The Two-Way ANOVA and Interactions between Treatments (Interaction Model)

As mentioned previously, there is a significant (*p* < 0.0001, α = 0.05) interaction between treatments T1 and T2 for all the observed parameters (RCT, k_20_, a_30_, a_60_, a_max_). The interaction model explains 99% of the total variation for the beginning of the coagulation (RCT, min). The interaction between treatments causes an extended time needed from rennet addition to the beginning of the coagulation (RCT, Figure 2a). As seen in Figure 2a, the RCT value rises, with the treatment concentration being the lowest for level 1 and the highest for the level 3 concentration treatment (T2). All three levels of T2 are significantly different (*p* < 0.0001, α = 0.05). Higher salt concentration extends the time needed for coagulation, while additional NaCl to KCl ratios still differ inside every investigated level (T2). 

The total variance explained by the interaction model for parameter k_20_ is 78% which is lower than for the other parameters but still good. As written by Bittante [15], the k_20_ parameter cannot be estimated for samples with high RCT value because curd firming does not attain 20 mm within 30 min, making it hard to compare it to a control sample. Nevertheless, the same author [15] states the tremendous practical importance of k_20_ as an indicator of the optimal time for curd cutting. The same trend of time elongation, as with RCT, is observed when looking at the interactions of k_20_ (Figure 2b). The time needed for the curd to reach a firmness of 20 mm is higher with every level of higher salt concentration. The lowest concentration (1%) had observed values closest to the control group, while the medium and highest concentrations (1.5% and 2%) had higher observed values for reaching CF of 20 mm. It is also seen that 100% NaCl or KCl also influenced the prolongation of time needed to reach CF of 20 mm, which will be discussed in Section 4.3 with one-way ANOVA results. According to Duncan’s MRT, level 1 is significantly different (*p* < 0.0001, α = 0.05) from levels 2 and 3, while levels 2 and 3 are not significantly different (*p* > 0.05) for the T2 treatment. Interaction between two treatments affects the increase in time needed to reach CF of 20 mm but only between low and medium/high concentrations. NaCl to KCl ratio seems to have a more substantial effect on k_20,_ which will be investigated in the linear model and discussed in the next section.

Curd firmness 30 min after rennet addition (a_30_) is highly dependent on the RCT and k_20_ values [15] because sometimes the time needed for the curd to reach 20 mm CF is longer than 30 min [19]. The interaction model in this study explained 98% of the total variance for this parameter. From the interaction figure (Figure 2c), the low level of salt concentration had values above the control, while the medium level of salt concentration had levels below the control values. The highest salt concentration (2%) had the lowest CF values 30 min after rennet addition. All three levels of salt treatment significantly affected (*p* < 0.0001, α = 0.05) the a_30_. This follows interactions in the parameters RCT and k_20_ because a low level of salt concentration had CF close and even higher than the control. In contrast, medium and high salt concentrations caused a delay in coagulation, so the curd did not have optimum firmness compared to the control after 30 min. 

They should be investigated together to discuss the parameters a_60_ and a_max_ properly. The total variance explained by the parameter a_60_ and a_max_ with the interaction model are 96% and 95%, respectively. The interactions between salt concentrations and different NaCl to KCl ratios are present. Figure 2d shows the interactions at 60 min (a_60_) after rennet addition, and it displays an opposite trend from the a_30_. Figure 2e shows interactions for maximum CF (a_max_). Following the slower beginning of the coagulation, the curd reaches its maximum CF values after 30 min (the control group had a_max_ at 24.50 min). The lower salt concentration resulted in faster coagulation and lower CF at the end of the analysis (a_60_) because it reached maximum CF between 30 to 40 min after rennet addition. The syneresis begins when the curd reaches maximum firmness (a_max_) [19]. Because of this, the curd firmness 60 min after rennet addition should be lower than the CF at 30 min. It shows optimal coagulation and syneresis processes.

### 4.3. The One-Way ANOVA of NaCl to KCl Ratio Effects (T1) inside Every Level of Treatment Concentration (T2), the Linear Model

According to Fox et al. [13], good curd forming properties represent rapid coagulation (low RCT value), high curd firming rate (low k_20_ value), and good curd firmness after a given renneting time (high a_30_ value). Teter et al. [54] published average MCP values for the Simmental breed of 17.15 ± 5.21 for RCT, 7.35 ± 3.19 for k_20_, and 21.78 ± 7.91 for a_30_, respectively. RCT value of the control group A1 (no salt added) was 10.00 ± 0.25; k_20_, 4.94 ± 0.66; a_30_, 17.89 ± 0.49; a_60_, 16.66 ± 2.53; and a_max_, 24.50 ± 0.80, respectively (Table 3).

The means, standard deviations, coefficients of determination, coefficients of variation, level of significance, and probability values of all T1 treatments inside every treatment T2 are shown in Table 3. Studies with the influence of NaCl on rennet coagulation and curd firming exist, but the effect of KCl on the same parameters is scarcely investigated.

RCT value was closest to the control (A, no salt) for C1 (1%, 50:50), C2 (1.5%, 50:50), and E3 (2%, 100% KCl), while B1-3 (1–2%, 100% NaCl) had the highest value. The presence of NaCl only significantly influences the primary enzymatic phase, prolongates the beginning of the coagulation, and is significantly different from all other treatments (A, C, D, E) inside every level of T2 (*p* < 0.001, α = 0.05), which is following other research [31,33]. Arora & Khetra [21] report that adding NaCl to milk causes longer coagulation time. Tsioulpas [34] reported a negative potassium correlation (−0.52) to RCT. Treatments with both NaCl and KCl are significantly different, only in a medium level of T2 (1.5%). In the lower T2 level (1%), D1 (25:75) and E1 (100% KCl), and in the higher T2 level (2.0%), C3 (50:50) and D3 (25:75) are not significantly different (*p* > 0.05). It can be concluded that NaCl has a greater impact on RCT than KCl, but the influence of group C (50:50, NaCl to KCl) is the closest to the control group A. 

The curd firming rate in the secondary phase of the coagulation process, or k_20,_ was also influenced by the different T1 treatments. In the lower level of the T2 treatment (1%), only B1 (100% NaCl) and E1 (100% KCl) were significantly different from the control A (*p* < 0.001, α = 0.05). C1 (1%, 50:50) and D1 (1%, 25:75) were close to control, with C1 being similar to A while D1 had a similar time to A and E1. It could imply that combining two salts in the reduced quantity (50:50 or 25:75) could better influence the curd firming rate in the lower salt level. In the medium level (1.5%), a significant difference from the control is observed (*p* < 0.001, α = 0.05) for all treatments. When looking at the differences between treatments, the 100% concentration of both salts had a similar effect on the curd firming rate, but their mixes (50:50 and 25:75) were significantly different (*p* < 0.001, α = 0.05). C2 (1.5%, 50:50) is nonsignificant to B2 (1.5%. 100% NaCl), D2 (1.5%, 25:75) is nonsignificant to B2 and E2 (1.5%, 100% KCl). For a 0.5% increase in salt concentration, treatment C (50:50) causes a curd firming rate closest to control A. In the highest level of salt concentration (2%), the treatments were significantly different from the control (*p* < 0.001, α = 0.05) but had no significant difference between them. High salt concentrations significantly slowed the curd firming rate regardless of the treatment. An overall conclusion is that through levels 1 to 3 of the T2, 100% of both salts had similar higher values for k_20_, while their mixes had moderate growth in curd firming rate through the rise of the salt concentration. Hatel & Reuter [55] report an increase in time for the curd to reach 20 mm with the addition of NaCl.

a_30_ marks the last coagulation phase after the curd structure formation [52]. It has an opposite trend to RCT. CF values (Table 3) are lowering, with the rise of the salt concentration being the highest at 1% and lowest at 2%. In level 1 (1%), treatments B1 (100% NaCl) and E1 (100% KCl) were closest to the control and similar, while treatments C1(50:50) and D1 (25:75) were significantly different from each other and all other treatments (*p* < 0.001, α = 0.05). The C1 treatment (50:50) had the largest CF value. In the medium salt concentration (1.5%), all the treatments had lower CF values than the control, with C2 (1.5%, 50:50) and E2 (1.5%, 100% KCl) being similar and B2 (1.5%. 100% NaCl) and D2 (1.5%, 25:75) being significantly different (*p* < 0.001, α = 0.05) from the control. In the highest salt concentration (2%), all the treatments were significantly different (*p* < 0.001, α = 0.05) from the control with low CF values. When looking at these values together with RCT (Table 3), it can be seen how coagulation started 1 to 4 min prior to this measurement while control started 20 min earlier. 

Salt concentrations above 1% influence curd firmness 30 min after rennet addition because extra time is needed to reach coagulation, thus not providing optimal CF in the given time.

a_60_ is used based on the research with prolonged observations [19] with difficulties in observing k_20_ and a_30_ to monitor the curd firming in slow coagulating milk. In the low salt concentration (1%), the curd firmness had the same trend as with a_30_. As shown in Table 3, treatments B1 (100% NaCl) and E1 (100% KCl) were similar and closest to the control; treatments C1 (50:50) and D1 (25:75) were significantly different from each other, and all other treatments (*p* < 0.001, α = 0.05). The C1 (50:50) had the largest CF value. In the medium level (1.5%), all treatments were significantly different from the control (A), D2 (25:75) was significantly different from other treatments (*p* < 0.001, α = 0.05), while B2 (100% NaCl), C2 (50:50), and E2 (100% KCl) were similar. The curd firmness was higher than the control because the slow coagulation resulted in higher values later than the control sample. In the highest salt concentration (2%), all treatments were again significantly different from the control (*p* < 0.001, α = 0.05) with the same trend as in a_30,_ which shows that all treatments had a steady rise in curd firmness which started late around 26 to 30 min. Treatments B3 (100% NaCl) and E3 (100% KCl) were significantly different from each other, but both mixes (50:50 and 25:75) were nonsignificant to them (*p* < 0.001, α = 0.05).

A_max_ is used to describe which treatment had the highest curd firmness. It is also the point after which the syneresis starts, and curd firmness falls due to the bursting of the curd and whey expulsion. In the lower salt concentration, (1%) treatments B1 (100% NaCl) and E1 (100% KCl) were similar and closest to the control (Table 3), while C1 (50:50) and D1 (25:75) were significantly different from the control and each other (*p* < 0.001, α = 0.05). The C1 (50:50) had the highest CF value. In the medium salt concentration (1.5%), C2 (50:50) had the highest CF, all the treatments were significantly different from the control, B2 (100% NaCl) and E2 (100% KCl) were not different from each other, C2 (50:50) and E2 (100% KCl) were also not differentiated and only D2 (25:75) was significantly different from all other treatments (*p* < 0.001, α = 0.05). In the highest salt concentration (2%), all the treatments were significantly different from the control (*p* < 0.001, α = 0.05), but no single treatment was differentiated from the others (Table 3). The E3 (100% KCl) had the highest CF.

## 5. Conclusions

A significant interaction (*p* < 0.001, α = 0.05) is present between the studied treatments T1 and T2. The rise in the salt concentration (T2) influences different NaCl to KCl ratios (T1) and their differences. An increase in salt concentration influences RCT and k_20_ by causing slower coagulation and curd firming rates.

The C1 (50:50) values (RCT, k_20_, a_30_, a_60_, a_max_) were closest to the control and had the best results of all T1 treatments in the lower (1%) and medium (1.5%) salt concentration. In comparison, in the highest salt concentration (2%), the treatment effect was nonsignificant (*p* < 0.001, α = 0.05). NaCl has a greater influence on MCP than KCl, but the influence of treatment C (50:50 NaCl to KCl) is the closest to control group A.

Salt concentrations above 1% influence curd firmness 30 min after rennet addition because extra time is needed to reach coagulation, thus not providing optimal CF in the given time. This makes it difficult to optimally consider k_20_, a_30_, a_60_, and a_max_ parameters and thoroughly investigate curd firming and syneresis. The salt concentration above 1.5% has a significant influence on the milk coagulation and MCP parameters (*p* < 0.001, α = 0.05) while complicating the observation of differences between the different NaCl to KCl ratios and their influence on the rennet coagulation and curd firmness. 

Future studies with a larger sample across multiple cattle breeds and different milk acidity values should be made. To bypass the deviations produced by late coagulating milk, which is a direct consequence of salt addition, the authors recommend prolonged observation and curd firmness modeling as a function of time explained by Bittante et al. [15]. These results should be a foundation for future studies investigating the influence of NaCl and KCl on the coagulation process and curd firmness to make cheese with lowered sodium appealing to consumers without losing quality.

## Figures and Tables

**Table 1:** Treatments with different NaCl to KCl ratios (T1).

T1	NaCl (%)	KCl (%)
A (CONTROL)	0	0
B	100	0
C	50	50
D	25	75
E	0	100

NaCl—sodium chloride; KCl—potassium chloride.

**Table 2:** Physicochemical and microbiological analyses of milk before adding treatments.

Parameters							
pH	Milk Fat (%)	Protein (%)	Lactose (%)	Total Solids (%)	Non-Fat Solids (%)	Somatic Cells Count/mL	TBC/mL
6.55	3.51	3.34	3.88	11.73	8.13	133,000	190,000

pH—milk acidity; TBC—total bacterial count.

**Table 3:** The results of the one-way ANOVA for treatments ratio (T1) in every salt concentration (T2) level.

T2		MCP				
	T1 Sample	RCT	k_20_	a_30_	a_60_	a_max_
1	A1	10.00 ± 0.25 ^a^	4.94 ± 0.66 ^a^	17.89 ± 0.49 ^a^	16.66 ± 2.53 ^a^	24.50 ± 0.80 ^a^
	B1	20.34 ± 0.31 ^b^	10.25 ± 3.42 ^b^	18.38 ± 1.67 ^a^	18.87 ± 1.67 ^a^	26.46 ± 3.92 ^a^
	C1	17.19 ± 0.52 ^c^	5.38 ± 0.75 ^a^	34.79 ± 2.33 ^b^	45.08 ± 2.12 ^b^	46.31 ± 1.23 ^b^
	D1	18.72 ± 0.29 ^d^	6.34 ± 0.60 ^ac^	28.91 ± 1.27 ^c^	26.71 ± 0.94 ^c^	34.55 ± 1.67 ^c^
	E1	18.97 ± 0.28 ^d^	9.75 ± 1.38 ^bc^	19.11 ± 2.33 ^a^	14.95 ± 1.67 ^a^	23.28 ± 0.94 ^a^
	R^2^	0.99	0.69	0.95	0.98	0.96
	CV	2.01	23.58	7.40	7.62	6.64
2	A2	10.00 ± 0.25 ^a^	4.94 ± 0.66 ^a^	17.89 ± 0.49 ^a^	16.66 ± 2.53 ^a^	24.50 ± 0.80 ^a^
	B2	25.44 ± 0.16 ^b^	8.81 ± 1.16 ^bc^	13.23 ± 1.70 ^bc^	36.75 ± 1.27 ^b^	37.24 ± 1.39 ^b^
	C2	22.75 ± 0.14 ^c^	7.31 ± 1.13 ^b^	15.93 ± 2.02 ^ab^	35.04 ± 2.70 ^b^	43.37 ± 2.17 ^c^
	D2	24.78 ± 0.12 ^d^	9.59 ± 0.98 ^c^	11.52 ± 2.17 ^c^	24.26 ± 3.70 ^c^	29.89 ± 2.33 ^d^
	E2	23.50 ± 0.20 ^e^	8.63 ± 1.01 ^bc^	16.42 ± 1.67 ^ab^	37.73 ± 3.96 ^b^	38.96 ± 3.14 ^bc^
	R^2^	0.99	0.78	0.71	0.91	0.93
	CV	0.85	12.75	11.45	9.93	6.11
3	A3	10.00 ± 0.25 ^a^	4.94 ± 0.66 ^a^	17.89 ± 0.49 ^a^	16.66 ± 2.53 ^a^	24.50 ± 0.80 ^a^
	B3	30.62 ± 0.35 ^b^	9.94 ± 1.25 ^b^	0.74 ± 0.94 ^b^	30.63 ± 1.67 ^b^	36.75 ± 1.70 ^bc^
	C3	29.00 ± 0.60 ^c^	10.00 ± 0.68 ^b^	2.21 ± 0.94 ^b^	32.83 ± 1.70 ^bc^	38.22 ± 1.13 ^bd^
	D3	29.16 ± 0.38 ^c^	10.16 ± 0.98 ^b^	4.17 ± 0.49 ^c^	34.06 ± 1.23 ^bc^	34.55 ± 1.47 ^c^
	E3	26.81 ± 0.52 ^d^	9.00 ± 0.87 ^b^	7.11 ± 0.94 ^d^	35.52 ± 1.47 ^c^	41.40 ± 2.02 ^d^
	R^2^	0.99	0.86	0.99	0.95	0.95
	CV	1.73	10.34	12.31	5.93	4.24

T1—treatment 1; T2—treatment 2; MCP—milk coagulation properties; RCT—rennet coagulation time; k_20_—time to reach curd firmness (CF) of 20 mm; a_30_—curd firmness 30 min after rennet addition; a_60_—curd firmness 60 min after rennet addition; a_max_—maximum curd firmness; the results are displayed as mean ($\overset{-}{X)}$ ± standard deviation (SD); N = 4—four observations per sample; R^2^—coefficient of determination; CV—coefficient of variation; different exponents in each column of the same treatment (T2) represent the statistical difference between observations (*p* > 0.0001, α = 0.05).

## Data Availability

The data supporting this study’s findings are available from the corresponding author, N.M., upon reasonable request.
